# A Novel Non-Cumbersome Approach Towards Biosynthesis of Pectic-Oligosaccharides by Non-Aflatoxigenic *Aspergillus* sp. Section Flavi Strain EGY1 DSM 101520 through Citrus Pectin Fermentation

**DOI:** 10.1371/journal.pone.0167981

**Published:** 2016-12-19

**Authors:** Amira M. Embaby, Ramy R. Melika, Ahmed Hussein, Amal H. El-Kamel, Heba S. Marey

**Affiliations:** 1 Institute of Graduate Studies and Research, Department of Biotechnology, University of Alexandria, Alexandria, Egypt; 2 Texas Tech University, Department of chemistry and Biochemistry, Lubbock, Texas, United States of America; 3 Faculty of Pharmacy, Department of Pharmaceutics, University of Alexandria, Alexandria, Egypt; 4 Institute of Graduate Studies and Research, Department of Environmental Studies, University of Alexandria, Alexandria, Egypt; 5 University of Alberta, Department of Civil and Environmental Engineering, Edmonton, AB, Canada; University of Szeged, HUNGARY

## Abstract

Pectic-Oligosaccharides (POS) have a growing potential in food and feed industries. To satisfy the demand of worldwide markets from POS and avoid the shortcomings of currently applied methodologies encountered in their preparation, the present study highlights a novel robust approach for POS biosynthesis. In the current approach, *Aspergillus* sp.section Flavi strain EGY1 DSM 101520 was grown on citrus pectin-based medium as a core POS production medium. POS' levels accumulated in the fungal fermentation broth were optimized through a three step sequential statistical mathematical methodology; Plackett-Burman design (PBD), Box-Behnken design (BBD) and canonical analysis. Three key determinants namely citrus pectin, peptone and NaH_2_PO_4_ were pointed out by PBD to impose significant consequences (P<0.05) on the process outcome (POS' levels). Optimal levels of these key determinants along with maximal of POS' levels were set by BBD and canonical analysis to be 2.28% (w/v) citrus pectin, 0.026% (w/v) peptone and 0.28% (w/v) NaH_2_PO_4_ to achieve a net amount of 1.3 g POS /2.28 g citrus pectin. Through this approach, a yield of 57% (w/w) POS of the total citrus pectin was obtained after 24 h of fungal growth on optimized citrus pectin–based medium. A fold enhancement of 13 times in POS' levels released in the fermentation fungal broth was realized by the end of the optimization strategy. This novel robust approach is considered a new insight towards POS biosynthesis via efficient, rapid and non-cumbersome procedure. To the best of authors' knowledge, the present work is the first article underlining detailed POS production from the fermentation broth of a fungus growing on citrus pectin-based medium.

## Introduction

Pectic-Oligosaccharides (POS) are indigestible pectin derived carbohydrates that might encompass oligogalacturonides (OGalA), galactooligosaccharides (GalOS), arabinooligosaccharides (AraOS), ramnogalacturonooligosaccharides (RhaGalAOS) and arabinogalactooligosaccharides (AraGalOS) [1]. In view of human health, they exhibit great therapeutic potentials as anti-cancer, anti-oxidant, anti-obesity, anti-bacterial and anti-ulcer as well as their confirmed robust functionalities as prebiotics [2–6]. The review of literature has displayed a set of technologies encountered in POS' preparation through de-polymerization of pectin-containing materials via pre-treatments methods viz. enzymatic, acidic and hydrothermal methods[6–9]. In enzymatic method, pectinases and non-pectinases enzymes are employed to act synergistically for better extraction of POS [6,10]. In spite enzymatic method is considered the most proper, safe and advantageous over acidic and hydrothermal ones for POS' preparation [11, 12], it is hampered by the relative high expense encountered in production and purification of such enzymes.

At most, pectin-containing materials that are good candidates for POS' preparation are pectin-containing feedstocks (e.g., orange peels, lemon peels, apple pomace, sugar beet pulp, etc) [13]. Processing of such materials for synthesis of POS is a multistep procedure, cumbersome, tedious, time consuming and probably expensive. These obstacles have triggered the scientific interest to continuously search for a rapid low cost effective and easily scalable bioprocess as an alternate to the currently applied methodologies for POS' synthesis.

Typically, fungal members belonging to *Aspergillus spp* are potential candidates to be employed in scalable bioprocesses for production of pectinases upon their growth on pectin-containing substrates. The by-product namely POS, normally released in the fermentation broth in a massive amounts due to the action of fungal pectinases on the pectin substrates during the growth-fermentation course, is completely negligible and non-efficiently utilized so far.

In the context of satisfying the worldwide markets form POS, the objective of the present study is to tailor a low cost effective efficient eco-friendly bioprocess for biosynthesis of POS by monitoring and optimizing the levels of these POS; normally accumulated by-product massively in the culture filtrate of *Aspergillus* sp.section Flavi strain EGY1 upon its growth on citrus pectin–based medium. To the best of our knowledge, the present work is the first article highlighting a detailed POS' production by the sake of the microorganism itself particularly a fungal strain not through an enzyme (pectinase)-substrate (purified citrus pectin) interaction; the most commonly applied method for POS' synthesis so far.

## Materials and Methods

### Chemicals

Citrus pectin(63–66% esterification) was purchased from LOBA Chemi Ltd. India. Peptone was obtained from Oxoid Co., UK. Agar agar was purchased from Titan Biotech Ltd., Delhi. Agarose. Rochelle salt, ethanol, glacial acetic acid and α-D-galacturonic acid were purchased from Sigma Co., USA. Dinitro-salicylic acid (DNSA) was purchased from LOBA Chemi Ltd. India.

### Fungal strain

*Aspergillus* sp. section Flavi strain EGY1 DSM 101520, a non-aflatoxigenic fungal strain, was used in this study during the course of POS' biosynthesis. This fungus was previously isolated and was identified by MALDI-TOF-MS and 18S rDNA technique (unpublished data). Moreover, 18S rDNA's nucleotide sequence of this fungus was deposited in the GenBank under the accession number of Gb: KM870530. While, the fungus was deposited in the open culture collection of DSMZ (Deutsche Sammlung von Mikroorganismen und Zellkulturen) and was assigned an accession number of DSM 101520. The inability of the fungus to produce aflatoxin was proved previously on a molecular level (unpublished data).

### Media

Potato dextrose agar [14] was used for handling and long term preservation of the fungal strain. Citrus pectin-based liquid medium (g/L: citrus pectin, 5.0 and peptone,5.0) was used in this study as an initial POS' core production medium. The final pH of the medium was adjusted to be 5.5.

### Testing pectin-degradative capability of the fungal strain

Pectinase activity of the fungal strain was tested upon its cultivation on citrus pectin–based agar medium for three days at 30°C. Pectinolytic capability of the fungus was pointed out by detecting hydrolytic zones around the fungal colonies after flooding the plate with 1% cetyl trimethylammonium bromide (CTAB) [15].

### Preparation of fungal seed culture

Twenty milliliters of citrus pectin-based liquid medium were inoculated with spores of the fungus collected from citrus pectin agar plate pre-grown for three days at 30°C. Then, this inoculated broth was incubated with an agitation speed of 150 rpm (New Brunswick incubator shaker, USA) at 30°C for two days.

### Estimation of POS' levels

POS' levels released in the fermentation broth during the course of fungal growth on citrus pectin-based liquid medium were determined in terms of reducing sugar as previously described [16]. Concisely, 0.5 mL of the cell free fermentation broth was added to 2 mL of dinitro-salicylic acid (DNSA). Then, boiling of this reaction mixture was performed for 10 minutes. Measuring of the developed color was performed at 540 nm (UV-Vis Spectronic Helios spectrophotometer, Thermo Scientific Co., USA) against blank (prepared similarly as the reaction except that water was added instead of cell free fermentation broth). A standard curve using α-D-galacturonic was established.

### Optimizing of POS' biosynthesis

Optimizing the process of POS' biosynthesis during the course of citrus pectin fermentation with the aid of *Aspergillus* sp. section Flavi strain EGY1 DSM 101520 was performed through a three step statistical-mathematical sequential approach; Plackett-Burman design, response surface methodology and canonical analysis.

### Plackett–Burman Design (PBD)

In Plackett-Burman designs [17], studying the impact of a set of independent variables on the process outcome could be carried out through a limited number of experimental runs as possible regardless the interactions between independent variables. Meanwhile, the linear effect compelled by N number of independent variables on a process output is estimated through a N+1 experiment. Typically, each independent variable is screened in two levels coded; +1 high level and -1 low level. Here, PBD was generated by Statistica software 12.0. Eight experimental runs with six independent variables (fractional factorial design) were conducted (Table 1). The following polynomial equation from the first order (Eq 1) was put in order to evaluate the linear effect imposed by the six tested independent variables on the level of reducing sugars as follow:

Y=βo+∑βixi (1)

Where *Y* is POS' level in terms of reducing sugars, *β*_*0*_ is the model intercept, *X*_*i*_ is the tested independent variable, namely citrus pectin, peptone, NaH_2_PO_4_, initial pH of the production medium, incubation temperature, and incubation time and *β*_*i*_ is the co-efficient of the tested independent variable. All experimental runs were conducted according to the PBD matrix in 250 mL Erlenmeyer flasks with working volume of 100 mL. All experimental runs were accomplished at agitation speed of 150 rpm (New Brunswick incubator shaker, USA). The initial levels of reducing sugars already existing in commercial citrus pectin or resulted from exposure of citrus pectin to autoclaving were omitted by determining the levels of reducing sugars at time zero after inoculation in each experimental trial. Then, these initial levels of reducing sugars were subtracted from the total levels of reducing sugars estimated at different time intervals after incubation in each experimental trial according to the design matrix. The average of the net of two readings of reducing sugars levels was taken for each experimental trial.

**Table 1:** Coded-Real levels of six independent variables in PBD for optimizing of POS' production by *Aspergillus* sp. section Flavi strain EGY1 DSM 101520 along with experimental and predicted levels of releasing POS.

Trial#	X1	X2	X3	X4	X5	X6	Y (Reducing sugars*, mg/mL)	
							Exp^a^,^c^.	Pred^b^.
**1**	-1	-1	1	-1	1	1	1.08975	1.059563
**2**	1	-1	1	1	1	-1	2.742	2.772188
**3**	-1	-1	-1	-1	-1	-1	1.782	1.812188
**4**	-1	1	-1	1	1	1	0.952	0.982188
**5**	1	1	-1	-1	1	-1	2.538	2.507813
**6**	1	-1	-1	1	-1	1	3.548	3.517813
**7**	1	1	1	-1	-1	1	1.582	1.612188
**8**	-1	1	1	1	-1	-1	0.12375	0.093563

-Values between in brackets are the real values of the independent variables. **X1:** citrus pectin (%w/v)**, X2:** peptone (%w/v), **X3:** NaH_2_PO_4_ (%w/v), **X4:** pH of the production medium, **X5:** incubation temperature (^o^C) and **X6:** incubation time (days)

* POS were determined in terms of reducing sugars

-a: experimental values &

b: predicted values and

c: net levels of reducing sugars after exclusion of any reducing sugars at time zero

### Box-Behnken Design (BBD)

Key determinants, pointed out through PBD with significant influences on the response in terms of POS' levels, were subjected to a response surface methodology in order to localize the optimal levels of these key determinants achieving the probable maximal POS' levels. Here, Box-Behnken design [18] was applied. Fifteen experimental trials for three independent variables were conducted (Table 2). As a rule, in BBD each independent variable is tested in three coded levels; -1 (low level), 0 (central level) and +1 (high level).

**Table 2:** Box-Behnken design with coded levels of three independent variables and experimental vs. predicted POS.

Trial#	X1	X2	X3	Y (Reducing sugars, mg/mL)*	
				Exp^a^,^c^.	Pred^b^.
**1**	-1	-1	0	0.214	0.974
**2**	1	-1	0	10.380	10.352
**3**	-1	1	0	0.480	0.508
**4**	1	1	0	4.390	3.630
**5**	-1	0	-1	0.377	0.075
**6**	1	0	-1	4.980	5.466
**7**	-1	0	1	0.180	-0.306
**8**	1	0	1	6.500	6.803
**9**	0	-1	-1	4.360	3.903
**10**	0	1	-1	0.360	0.634
**11**	0	-1	1	4.980	4.706
**12**	0	1	1	0.330	0.787
**13**	0	0	0	0.310	0.337
**14**	0	0	0	0.3100	0.337
**15**	0	0	0	0.39	0.337

a: Experimental values

b^:^ predicted values and

c: net levels of reducing sugars after exclusion of any reducing sugars at time zero

X1: citrus pectin, X2: peptone and X3: NaH_2_PO_4_

*POS were determined in terms of reducing sugars/mL

The following polynomial equation of the second order (Eq 2) was set in order to outline the influence of all probable forms of interactions assuming to be enforced by the independent variables on POS' levels liberated from citrus pectin during the course of fungal growth on citrus pectin–based liquid medium.

$$Y=\beta o+\sum_{i=1}^{k}\beta ixi+\sum_{i=1}^{k}\beta iixixi+\sum_{i=1}^{k-1}\sum_{j=2}^{k}\beta ijxixj+\epsilon$$ (2)

Where *Y* is the level of liberated POS in terms of reducing sugars (response), *x*_*1*_, *x*_*2*_, *x*_*3*,*………*_*x*_*k*_ are the independent variables affecting the response, *β*_*0*_ is the model intercept,*βi*(i = 1, 2,…, k) is the linear estimate of the variable, *βii* (i = 1, 2,…, k) is the quadratic estimate of the variable, *βij* (i = 1, 2,…, k; *j* = 1, 2,…, k) is the cross interaction estimate of the variable and € is the random error. For statistical calculations, each independent variable *X* was coded as *Xi* according to the Eq 3.

$$X_{i}=(xi-x_{o})/\Delta xi$$ (3)

Where *X*_*i*_ is dimensional coded value of the independent variable, *xi* is the real value of this variable at this coded value, *x*_*o*_ is the real value of this variable at the center point (zero level) and *Δxi* is the step change value. All experimental runs were conducted according to the Box–Behnken matrix in 250 mL Erlenmeyer flasks with a working volume of 100 mL. All experimental runs were carried out at agitation speed of 150 rpm. The initial levels of reducing sugars already existing in commercial citrus pectin or resulted from exposure of citrus pectin to autoclaving were omitted by determining the levels of reducing sugars at time zero after inoculation in each experimental trial. Then, these initial levels of reducing sugars were subtracted from the total levels of reducing sugars estimated at different time intervals after incubation in each experimental trial according to the design matrix. The mean of the net of two readings of reducing sugars levels was taken for each experimental trial.

### Statistical and canonical analyses

Statistica software 12.0 was employed to create PBD-BBD matrices, carry out multiple linear and non-linear regression analyses and draw the three dimensional surface plots. Canonical analysis was performed via RSM package (R development Core team 2016), available from the Comprehensive R Archive Network (http://CRAN.R-project.org/package=rsm).

## Results

### Pectinolytic capability of *Aspergillus* sp. section Flavi strain EGY1 DSM 101520

The capability of *Aspergillus* sp. section Flavi strain EGY1 DSM 101520 toi hydrolyze citrus pectin was proved through visualizing a zone of hydrolysis surrounding the fungal colonies growing on citrus pectin agar plate (data not shown) after three days of incubation at 30°C.Moreover, POS' levels were monitored during the course of fungal growth-fermentation on citrus pectin–based liquid medium along for four successive days and a perceivable POS' level (1 mg/mL) was obtained after 24 h (data not shown). After 24 h, a decline in POS' levels was noticed (data not shown).

### Statistical-mathematical Optimization of POS' biosynthesis by the fungal strain

#### Screening key determinants by PBD

For PBD, the experimental values vs. the predicted values of the process outcome (POS) were illustrated in Table 1. Experimental results for net POS' levels varied from 0.124 to 1.782 mg/mL reducing sugars that in turn conferred the indispensable need to carry out this step in the optimization plan. Where, the results of linear multiple regressions of data were pointed out in Table 3. F and P-values of the model were 195.72 and 0.0546, respectively as elucidated from ANOVA results. This F-value pointed out the model' significance. Whereas, the P-value indicated that the likelihood is only 5.4% that this model F-value could happen due to noise. An independent variable having a co-efficient at a P- value ≤0.05 was selected to impose a significant influence on the process output. Multiple regression of data in PBD revealed that there exist three independent variables (pectin, peptone and NaH_2_PO_4_) provoking significant consequences (P≤0.05) on POS production. Only one independent variable (citrus pectin) out of the three independent variables (citrus pectin, peptone and NaH_2_PO_4_) imposed positive significance on the process outcome whilst, the other two remaining independent variables (peptone and NaH_2_PO_4_) showed significant negative impact. A first order polynomial Eq (4) in terms of coded values was established to explain the linear relation between the independent variables and the process outcome as follows:

Y=1.79+0.807X1−0.496X2−0.41X3+0.047X4+0.034X5−0.0018X6 (4)

**Table 3:** Real-coded values of independent variables and regression analysis for POS' optimization by PBD.

Independent Variable	Low level -1	High level +1	Main effect	*B*-coefficient	P-value	t-value	% confidence
**Citrus Pectin** **(X1**, w/v %)	0.2	0.6	1.62	0.807813	0.023779*	26.75983	97.60*
**Peptone** (**X2**, w/v %)	0.0	0.5	-0.992	-0.49575	0.038718*	-16.4224	96.13*
**NaH**_**2**_**PO**_**4**_ (**X3**, w/v %)	0.0	0.1	-0.82	-0.41031	0.046753*	-13.5921	95.33*
Initial pH of the medium(X4)	4.0	6.0	0.094	0.04675	0.365014	1.548654	63.49
Incubation temperature (X5,°C)	30	37	0.072	0.03575	0.446421	1.184265	55.36
Incubation time (X6, days)	1.0	5.0	-0.004	-0.00175	0.963136	-0.05797	3.69

*Significant P-value ≤ 0.05 & R^2^ = 0.99 & Adjusted R^2^ = 0.99 and P -value for the model = 0.05

Other independent variables (e.g., incubation temperature, incubation time and initial pH of the production medium) showing non-significant consequence on the process outcome were set either at their lowest values or at intermediate values between the low value and the high value. Based on this conclusion derived from PBD, next experiments were conducted at 30°C, pH (5.0) for 24 h.

#### Localizing optimal levels of key determinants

These three key determinants (citrus pectin, peptone and NaH_2_PO_4_) were subjected to response surface methodology in order to localize the optimal level of each key determinant along with the maximal level of the process output (POS). Herein, Box-Behnken design was applied. The experimental values vs. the predicted values of POS were shown in Table 2. Coded–real values of the tested independent variables along with results of multiple non- linear regression were demonstrated in Table 4. F-value and P-value were 32.03 and 0.00067, respectively. These values implied the significance of the model and the chance (0.067%) that this F-value might take place due to noise. Model aptness is evidenced by the value of R^2^ (0.98) that conferred the good agreement between the experimental and the predicted values of the response.

**Table 4:** Real–coded values of independent variables and regression analysis for POS optimization by Box-Behnken.

Variable	Low level -1	Middle level 0	High level +1	Model term	Main effect	*B-*coefficient	t-value	P-value	% confidence
**Citrus Pectin (X1, %w/v)**	0.2	1.2	2.2	X1	6.24975	3.124875	12.79921	5.18E-05*	99.99*
**Peptone (X2, %w/v)**	0.05	0.65	1.25	X2	-3.5935	-1.79675	-7.35933	0.000727*	99.99*
**NaH**_**2**_**PO**_**4**_ **(X3, %w/v)**	0.02	0.22	0.42	X3	0.47825	0.239125	0.979435	0.372346	62.7654
				X1X1	4.031084	2.015542	5.608495	0.002492*	99.75*
				X2X2	3.027584	1.513792	4.212313	0.00839*	99.161*
				X3X3	1.314084	0.657042	1.8283	0.127045	87.2955
				X1X2	-3.1280	-1.56400	-4.52973	0.006227*	99.3773*
				X1X3	0.85850	0.42925	1.243214	0.268904	73.1096
				X2X3	-0.3250	-0.1625	-0.47064	0.657714	34.2286

*Significant P-value <0.05 & R^2^ = 0.98& Adjusted R^2^ = 0.95 and P -value for the model = 0.000677

A second order polynomial Eq 5 was confined in terms of coded values to illustrate all likely interactions among the tested independent variables that might enforce an influence on the response.

Y=0.336+3.12X1−1.79X2+0.239X3+2.01X1.X1+1.51X2.X2+0.657X3.X3−1.564X1.X2+0.429X1.X3−0.163X2.X3 (5)

Based on data derived from multiple non-linear regression, citrus pectin imposed significant consequences (P<0.05) on the process outcome in three forms, linear, quadratic and cross interaction with peptone (Table 4). However, peptone imposed significant influence on the response in two forms: linear and quadratic interactions. Whilst, no significant effectuations could be traced by NaH_2_PO_4_. With regard to factors imposing significant effect on the response, the results of multiple non-linear regression for BBD are in a good agreement with those derived from multiple linear regression derived from PBD.

#### Attaining optimized conditions for maximal response

To attain the optimized conditions for the maximal response, Eq 5 was differentiated. The obtained predicted stationary point has the predictor's combination set in terms of coded values: X1 = -0.689, X2 = 0.241 and X3 = 0.073 to achieve POS' level of -0.948 mg/mL. This predicted stationary point is an unrealistic minimum stationary point. This addressed the indispensable need to carry out canonical analysis to localize the realistic stationary point achieving the maximal response. Canonical analysis is a key analysis as it verifies whether the predicted stationary point is a saddle, maximum or minimum. The shape of the response could be made certain by eigen-values and eigenvectors of the matrix of second order. Eigenvectors could delimit the direction of principle orientation of the surface. Where, signs and magnitude of eigen-values are real analytic of surface shape in these directions. Theory of eigen–values and their mathematical designations were elucidated in details earlier in two rules of Myers (1976) [19]. Downward and upward curvature of the response could be deduced from negative and positive eigen-values, respectively (1^st^ rule of Myers). However, the magnitude of an eigen–value in its absolute value determines that the response curvature would be in the associated direction (2^nd^ rule of Myers). The present model of BBD with three independent variables exhibited the following eigen-values: λ_1_ = 2.611, λ_2_ = 0.955 and λ_3_ = 0.621. These positive eigen-values deduced that the predicted stationary point is a minimum (upward curvature) neither a maximum nor a saddle one. The large value of these eigen-value, that was for X1 (2.611, indicated that the response curvature would be associated with this direction. In canonical analysis, the stationary point was traced starting from the predicted unrealistic stationary point by moving along the canonical path in both directions as illustrated in Table 5.

**Table 5:** Estimated maximum response (POS) production by *Aspergillus* sp. section Flavi strain EGY1 DSM 101520 using a canonical path.

Distance	Coded values of independent variables			Estimated response Y POS (mg/mL)
	X1	X2	X3	
-1.00	-1.495	0.823	-0.040	1.663
-0.8	-1.334	0.707	-0.0.17	0.724
-0.6	-1.172	0.591	0.005	-0.009
-0.4	-1.011	0.474	0.028	-0.532
-0.2	-0.850	0.358	0.051	-0.844
**0.0**	**-0.689**	**0.241**	**0.073**	**-0.948**
0.2	-0.528	0.125	0.096	-0.844
0.4	-0.367	0.008	0.118	-0.530
0.6	-0.206	-0.108	0.141	-0.008
0.8	-0.045	0.225	0.163	0.724
1.0	0.116	-0.41	0.186	1.664
1.2	0.277	-0.457	0.208	2.811
1.4	0.438	-0.574	0.231	4.171
1.6	0.599	-0.690	0.253	5.735
1.8	0.760	-0.807	0.276	7.513
2.0	0.921	-0.923	0.298	9.494
2.1	1.002	-0.981	0.310	10.568
2.2	1.082	-1.040	0.321	11.691
2.3	1.163	-1.098	0.332	12.869
2.4	1.243	-1.156	0.343	14.089

Bold values represent the predicted stationary point derived from differentiating of the 2^nd^ polynomial equation

**Fig 1:**
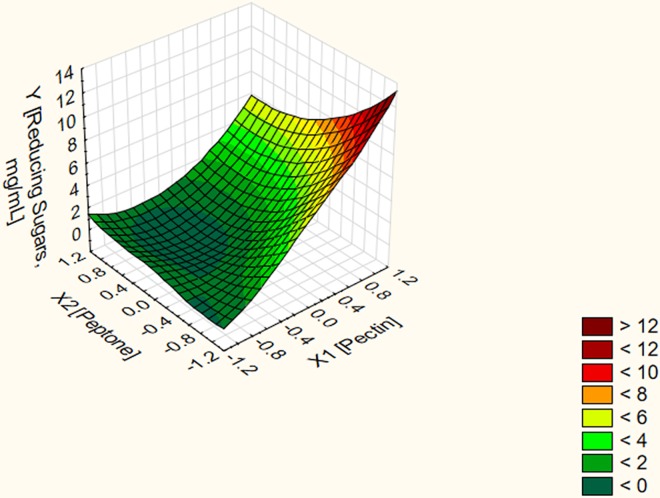
Three dimensional surface plot for the dependent variable POS in terms of reducing sugars vs. the independent variables pectin and peptone at constant optimal value of NaH_2_PO_4_.

**Fig 2:**
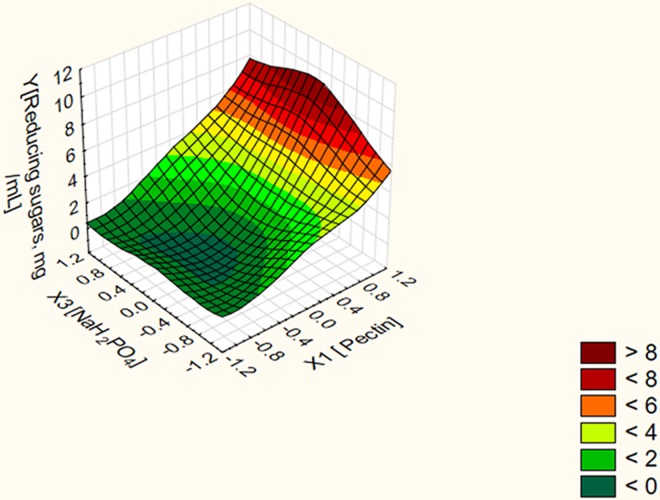
Three dimensional surface plot for the dependent variable POS in terms of reducing sugars vs. the independent variables pectin and NaH_2_PO_4_ at constant optimal value of peptone.

**Fig 3:**
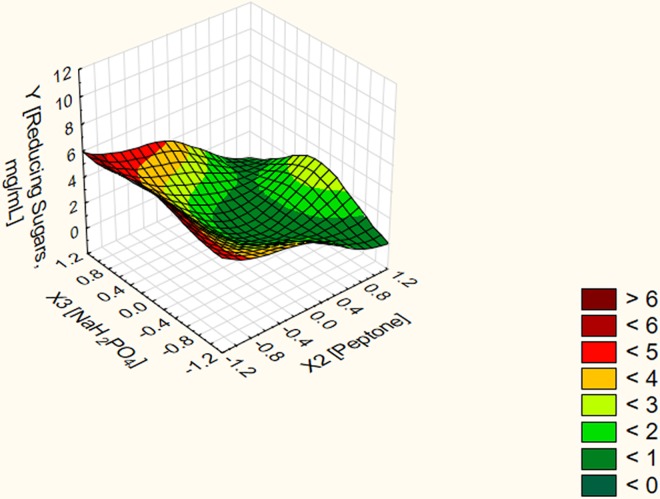
Three dimensional surface plot for the dependent variable POS in terms of reducing sugars vs. the independent variables peptone and NaH_2_PO_4_ at constant optimal value of pectin.

On the other hand, the three dimensional surface plots verified that the predicted stationary point is minimum one with downward curvature (Figs 1–3). Moving in the negative direction of the canonical path has resulted in obtaining unrealistic stationary points as shown in Table 5. Conversely, moving along the canonical path in the positive direction at a distance of 0.8 and above resulted in obtaining different predictor's combination sets corresponding to a stepwise increase in the response without reaching a stationary point. To localize the optimal conditions, the last four predictor's combination sets at distances 2.1, 2.2, 2.3 and 2.4 were validated experimentally in the lab. Experimental data revealed that using the predictor's combination set (X1 = 1.082, X2 = -1.04 and X3 = 0.321) at a distance 2.2 resulted in a net POS' level of 13 mg/mL. Using the predictor's combination set (X1 = 1.002, X2 = -0.981 and X3 = 0.310) at a distance 2.1 resulted in a net POS' level of 11 mg/mL. However, no further increase was obtained upon using the other two predictor's combination sets at distances 2.3 and 2.4 in the canonical path. Thus, the predictor's combination set in terms of real values (X1 = 2.28% (w/v), X2 = 0.026% (w/v) and X3 = 0.28% (w/v)) at a distance 2.2 resulted in a net POS' level of 13 mg/mL. Experimental data conferred model adequacy and precision since the percent of validation was 100%. In addition, the net yield of the bioprocess for POS biosynthesis is 1.3 g POS/2.28 g citrus pectin. In other words, it represents approximately 57% POS (w/v) of citrus pectin as initial materials. A fold enhancement of 13 times for POS' yield released in the fungal fermentation broth was achieved by the end of the optimization strategy.

## Discussion

Fungal members as pectinase producers are proper candidates for scalable pectinase production bioprocess regarding the high yield obtained comparing to that obtained from bacterial pectinase producers. The issue concerned with production, optimizing and characterizing such pectinases of microbial origin has been covered thoroughly in several researches worldwide due to their involvement in numerous biotechnological applications [20–33].So far, no articles in the review of literature handle possible utilization of POS, negligible by-products that release massively in the fermentation broth of these fungal members growing on citrus pectin-based medium. In addition, currently applied methodologies in scalable processes for POS' biosynthesis include shortcomings like high expense of enzymes encountered in their preparation and difficulties of raw pectin-containing materials' processing. Additionally, the demand for bioactive POS particularly in biomedical, food and feed industries is growing nowadays. In this context, a pectinase producer fungal member belonging to the genus *Aspergillus* was selected to carry out the present study aiming to biosynthesize POS with the aid of the microorganism itself.

Preliminary results concerning monitoring of POS' levels (1 mg/mL) released in the fermentation broth during the growth of *Aspergillus* sp. section Flavi strain EGY1 DSM 101520 on citrus pectin- based medium after 24 h greatly elicited the authors' attention to optimize the production of these POS. As a rule, in bioprocess optimization a number of objectives should be achieved at last. These objectives are confined to low substrate consumption, high product yield, high process reliability and reproducibility, efficient unit operation, high product quality, non-cumbersome downstream and upstream processing, low overall cost of entire bioprocess and efficient process scheduling. Prior to the transfer to the industrialized commercialization step, the yield of a bioprocess output should be made great as much as possible. As a consequence, optimizing the yield of an outcome of a bioprocess has aggravated the scientific interest of many operators. In the interim, the transfer from the shake flask scale to the industrial scale is greatly hampered by the bioprocess' capital cost. Even so, the cost of production medium stands for 30–40% of the capital expenditure of any bioprocess [33]. Therefore, customizing of a cheap and efficient production medium that highly triggers the microbe towards bulk production of the target end product is a defy encountered in upstream processing. Herein, a three steps sequential statistical-mathematical approach was employed to maximize the level of produced POS upon growing of *Aspergillus* sp. section Flavi strain EGY1 DSM 101520 on citrus pectin–based medium.

Statistical designs (e.g., full/fractional factorial designs and response surface methodology) are experimental designs that are anticipated for process optimization with regard to screening the process key determinants and localizing the optimal levels of these key determinants in conjunction with the process output (response). These designs have been employed for maximizing the yield of enzymes, soluble proteins, amino acids, antimicrobial agents, etc [31,34,35] instead of the traditional methods (one variable at a time).

The optimized core POS' production medium utilized in the present study contained only three components namely, citrus pectin, peptone and NaH_2_PO_4_.The last two components were set at very minute amounts: 0.026 and 0.28% (w/v), respectively. Although, citrus pectin is relatively of intermediate cost, it is incorporated in the core POS production medium in a relatively low concentration. This in turn would reduce the capital cost of the production medium. As a rule of thumb, minimizing the number of steps required during upstream processing and downstream processing would greatly underpin diminishing the total capital cost and gaining non-cumbersome bioprocesses upon scalability. Although medium cost stands for 30–40% of the total capital cost [33], but certain issues related to the substrate used in the core production medium should be taken into account. These substrates related issues could greatly impose negative or positive consequences on the execution of a given bioprocess on the industrial scale. These issues are mostly confined to substrate type (e.g., waste, raw, purified byproduct, etc), substantial variations in composition from batch to batch, availability all the year and requirement for mechanical and/or physical treatment for a raw substrate prior its addition to the core production medium. At most, substrates with low degree of purity (e.g., waste, raw materials, etc) would address a multistep procedure in downstream processing for refining, purifying and recovery of the target product that would in turn add an additional cost to the total capital cost particularly for bio-products demonstrating applications in medicine and food industries. Raw substrates (e.g., agro-industrial orange peel waste, lemon peel waste, etc) would require physical and/or mechanical treatments to make them accessible for the producers (i.e., fungi) in upstream processing that would in turn add an additional cost to the total capital cost. From the standpoint of reproducibility and validity of the obtained results, highly purified substrates (e.g., commercial purified citrus pectin) would be advantageous over than raw agro-industrial wastes regarding consistency in composition among different batches of the substrate. Furthermore, the low initial concentration of citrus pectin (2.28%w/v), as a starting material to biosynthesize the POS by *Aspergillus* sp. section Flavi strain EGY1 DSM 101520 would participate also in reducing the viscosity of the production medium. This reduced viscosity would result in non-cumbersome duties encountered in both upstream and downstream processing. In the light of advantages of using purified substrates (e.g., purified commercial citrus pectin) over using raw substrates as discussed above, present finding is superior to other findings reported in the literature stating that 100 g of lemon peels and 9 kg of orange albedo waste, are required as starting pectin materials in the course of POS' preparation via enzymatic and acidic methods [1, 36].Definitely, using these agro-industrial wastes (lemon peels and albedo waste) as substrates would encounter all the aforementioned obstacles derived from using substrate -based wastes. An appreciable net yield of 1.3 g of POS/2.28 g of citrus pectin as in initial material was obtained. It constitutes 57% (w/w) POS of the total citrus pectin as a starting material. This yield is relatively high when compared to others previously reported in the literature. For instance, orange peel hydrothermally pretreated followed by enzymatic processing resulted in a yield of 25.1% (w/v) of the total oven dried raw material [7].Whereas, sugar beet pulp (SBP) hydrothermally treated followed by enzymatic processing generated a yield of 31.2% (w/v) POS of the total oven dried SBP [37].Conversely, the present finding regarding the yield of POS was slightly lower than the yield of POS (81% (w/w) from passion fruit pectin [38].Interestingly, a fold enhancement of 13 in POS' levels was achieved by the end of the optimization strategy. This would in turn verify the conclusion stating that optimizing the POS' levels in the present approach was a mandatory task prior scalability of this bioprocess. So far, the literature of review contains a very few number of articles highlighting detailed statistical optimization for the process of POS' biosynthesis even using the current applied methodologies like enzymatic method [38, 39].

Present results reveal that the bioprocess could be conducted well at 30°C with production of satisfactory POS' levels. This low temperature required for POS' synthesis by *Aspergillus* sp. section Flavi strain EGY1 DSM 101520 is advantageous from the standpoint of energy savings. On the contrary, in majority of acidic and hydrothermal methods high temperature greater than 121°C is a prerequisite of completion of pectin hydrolysis to release POS [14, 40, 41]. Even so, enzymatic hydrolysis of pectin containing materials should be performed under temperature degree at least not less than 37°C [1, 36, 38, 42].

With regard to the time factor as a key factor in the platform of a commercialized bioprocessing, the lower the cost would be imposed for a bioprocess as long as the duration time required for accomplishing of such bioprocess is shortened. Present data reveal that appreciable levels of the target product POS (1.3 g POS/2.28 g citrus pectin) could be reached after 24 h of cultivation of *Aspergillus* sp. section Flavi strain EGY1DSM 101520 on optimized citrus pectin-based medium. Present data is partially in agreement with others reported in the literature where duration times ranged from very few hours to 48h in order to accomplish acidic or enzymatic pectin hydrolysis [14, 38, 40, 42].

Present approach displays certain privileges over the traditional alternates. These privileges could be outlined by low cost of the production medium, time saving, energy saving, unrestricted availability of conducting bulky preparation of POS on an industrial scale, reproducibility, reliability, safety of the bioprocess conducted by non-aflatoxigenic *Aspergillus* sp. section Flavi strain, no liberation of toxic byproducts posing potential environmental hazards, and concomitant production of industrially important fungal enzyme (i.e., pectinase).

## Conclusions

The present approach is considered a new insight towards biosynthesis of POS, a negligible by-product in the fungal citrus pectin fermentation broth with high potential in medical-industrial sectors. The present work encounters two aspects of novelty mainly confined to a) using the whole microbial cells (i.e., the fungal strain itself) not part of it (i.e., pectinases alone) to prepare POS and b) optimizing of POS' levels through robust statistical–mathematical designs. By applying the present approach, a considerable level of POS was achieved. In this context, present data would greatly underpin the potential of the present approach as a promising alternate for the currently applied methodologies for POS' preparation regarding low cost-effectiveness, reproducibility, reliability, energy saving, non-cumbersomeness and considerable yield. Future work will focus on thorough separation and characterization of these POS for judging its suitability in bioassays prior the industrialized commercialization stage.

## References

[1] Gomez B, Yaez RR, Parajo JC, Alonso JL. Production of pectin-derived oligosaccharides from lemon peels by extraction, enzymatic hydrolysis and membrane filtration. J Chem Technol Biotechnol.2014;

[2] Rastall RA, Gibson GR, Gill HS, Guarner F, Klaenhammer TR, Pot B, et al Modulation of the microbial ecology of the human colon by probiotics, prebiotics, prebiotics and synbiotics to enhance human health: An overview of enabling science and potential applications. FEMS Microbiol Ecol.2005; 52:145–152. 10.1016/j.femsec.2005.01.003 16329901

[3] Olano-Marton E, Rimbach GH, Gibson GR and Rastall RA. Pectin and pectic-oligosaccharides induce apoptosis in *in vitro* human colonic adenocarcinoma cells. Anticancer Res. 2003;23:341–346. 12680234

[4] Holck J, Hjerno K, Lorentzen A, Vigsnaes LK, Hemmingsen L, Licht TR, et al Tailored enzymatic production of oligosaccharides from sugar beet pectin and evidence of differential effects of a single DP chain length difference on human faecal microbiota composition after *in vitro* fermentation. Process Biochem.2011; 46:1039–1049.

[5] Gullon B, Gomez B, Martonez-Sabajanes M, Yanez R, Parajo JC, Alonso JL. Pectic oligosaccharides: manufacture and functional properties. Trends Food Sci Technol. 2013; 30:153–161.

[6] Babbar N, Dejonghe W, Gatti M, Sforza S, Elst K. Pectic oligosaccharides from agricultural by-products: production, characterization and health benefits. Crit Rev Biotechnol. 2016; 36(4):594–606. 10.3109/07388551.2014.996732 25641325

[7] Martinez M, Yanez R, Alonso JL, Paragó JC. Chemical production of pectic oligosaccharides from orange peel wastes. Ind Eng Chem Res. 2010;49: 8470–8476.

[8] Chen J, Liang RH, Liu W, Li T, Liu CM, Wu SS, et al Pectic-oligosaccharides prepared by dynamic high-pressure microfluidization and their *in vitro* fermentation properties. Carbohydr Polym. 2013; 91: 175–182. 10.1016/j.carbpol.2012.08.021 23044120

[9] Combo AM, Aguedo M, Quievy N, Danthine S, Goffin D, Jacquet N, et al Characterization of sugar beet pectic-derived oligosaccharides obtained by enzymatic hydrolysis. Int J Biol Macromol. 2013; 52: 148–156. 10.1016/j.ijbiomac.2012.09.006 22986181

[10] Pedrolli DB, Monteiro AC, Gomes E, Carmona EC. Pectin and pectinases: production, characterization and industrial application of microbial pectinolytic enzymes. The Open Biotechnol J. 2009; 3: 9–18.

[11] Dagnino EO, Roggero Luque FS, Morales WG, Chamorro ER, Felissia FE, Area MC, et al. Hidrolisis enzimatica de cascarilla de arroz pre- tratada con ácido diluido para evaluar la eficacia de la etapa de pretratamiento. II Jornadas de Investigaciَn en Ingenierea del NEA y paoses limatrofes, “Hacia donde van la ciencia y la tecnologia en el MERCOSUR” Resistencia, Argentina (2009).

[12] Zhu L. Fundamentals study of structural features affecting enzymatic hydrolysis of lignocellulosic biomass. PhD. Thesis, Texas A&M University. 2005.Available from: http://oaktrust.library.tamu.edu/handle/1969.1/4314?show=full

[13] Gómez B, Gullón B, Remoroza C, Schols HA, Parajó JC, Alonso JL. Purification, Characterization, and Prebiotic Properties of Pectic Oligosaccharides from Orange Peel Wastes. J Agric Food Chem. 2014; 62:9769−9782. 10.1021/jf503475b 25207862

[14] Downes FP, Ito K. (Eds.). Compendium of Methods for the Microbiological Examination of Foods, 4th Ed., APHA, Washington, D.C; 2001.

[15] Hadj-Taieb N, Tounsi H, Chabchoub A, Abid N, Gargouri A. Studies on the zymogram method for the detection of pectinolytic activities using CTAB. Appl Biochem Biotechnol. 2011; 165(7–8):1652–60. 10.1007/s12010-011-9384-y 21935586

[16] Miller GL. The use of dinitrosalicylic acid reagent for the determination of reducing sugars. Anal Chem. 1959; 31: 426–428.

[17] Plackett RL, Burman JP. The design of optimum multifactorial experiments.Biometrica.1946; 37:305–325.

[18] Box GEP, Behnken DW. Some new three level design for study of quantitative variables. Technometrics.1960; 2:455–475.

[19] Myers RH. Response Surface Methodology. Edwards Brothers, Ann Arbor, MI;1976.

[20] Mohamed SA, Al-Malki AL, Khan JA, Kabli SA, Al-Garni SM. Solid state production of polygalacturonase and xylanase by *Trichoderma* species using cantaloupe and watermelon rinds. J Microbiol. 2013; 51(5):605–611. 10.1007/s12275-013-3016-x 24037654

[21] Maller A, Damásio AR, da Silva TM, Jorge JA, Terenzi HF, Polizeli Mde L. Biotechnological Potential of Agro-Industrial Wastes as a Carbon Source to Thermostable Polygalacturonase Production in *Aspergillus niveus*. Enzyme Res.2011; 2011:289206 10.4061/2011/289206 21837272 PMC3132474

[22] Demir H, Tari C. Effect of physicochemical parameters on the polygalacturonase of an *Aspergillus sojae* mutant using wheat bran, an agro-industrial waste, via solid-state fermentation. J Sci Food Agric.2016; 96(10):3575–3582. 10.1002/jsfa.7543 26604188

[23] Martin N, Guez MA, Sette LD, Da Silva R, Gomes E. Pectinase production by a Brazilian thermophilic fungus *Thermomucor indicae*-seudaticae N31 in solid-state and submerged fermentation.Mikrobiologia.2010; 79(3):321–328. 20734812

[24] Pedrolli DB, Carmona EC. Purification and Characterization of a Unique Pectin Lyase from *Aspergillus giganteus* Able to Release Unsaturated Monogalacturonate during Pectin Degradation. Enzyme Res.2014;353915 10.1155/2014/353915 25610636 PMC4294307

[25] Xu SX, Qin X, Liu B, Zhang DQ, Zhang W, Wu K, Zhang YH. An acidic pectin lyase from *Aspergillus niger* with favorable efficiency in fruit juice clarification. Lett Appl Microbiol.2015; 60(2):181–187. 10.1111/lam.12357 25382689

[26] Mata-Gómez MA, Heerd D, Oyanguren-García I, Barbero F, Rito-Palomares M, Fernández-Lahore M.A novel pectin-degrading enzyme complex from *Aspergillus sojae* ATCC 20235 mutants. J Sci Food Agric.2015; 95(7):1554–61. 10.1002/jsfa.6864 25103563

[27] Sohail M, Latif Z. Phylogenetic Analysis of Polygalacturonase-Producing *Bacillus* and *Pseudomonas* Isolated From Plant Waste Material. Jundishapur J Microbiol.2016; 9(1):e28594 10.5812/jjm.28594 27099686 PMC4834142

[28] Rehman HU, Qader SA, Aman A.Polygalacturonase: production of pectin depolymerizing enzyme from *Bacillus licheniformis* KIBGE IB-21.Carbohydr Polym. 2012; 90(1):387–391. 10.1016/j.carbpol.2012.05.055 24751056

[29] Tamburini E, León AG, Perito B, Mastromei G. Characterization of bacterial pectinolytic strains involved in the water retting process. Environ Microbiol. 2003;5(9):730–736. 12919408 10.1046/j.1462-2920.2003.00462.x

[30] Irshad M, Asgher M, Anwar Z, Ahmad A. Biotechnological valorization of pectinolytics and their industrial applications: a review. Nat Prod Commun. 2014; 9(11):1649–1654. 25532302

[31] Embaby AM, Masoud AA, Marey HS, Shaban NZ, Ghonaim TM. Raw agro-industrial orange peel waste as a low cost effective inducer for alkaline polygalacturonase production from *Bacillus licheniformis* SHG10.SpringerPlus.2014; 3:327 10.1186/2193-1801-3-327 25077057 PMC4112199

[32] Tepe O, Dursun AY. Exo-pectinase production by *Bacillus pumilus* using different agricultural wastes and optimizing of medium components using response surface methodology. Environ Sci Pollut Res Int. 2014;21(16):9911–20. 10.1007/s11356-014-2833-8 24819433

[33] Bayoumi RA YH, Swelim MA, Abdel-All EZ. Production of Bacterial Pectinase(s) from Agro-Industrial Wastes Under Solid State Fermentation Conditions. J Appl Sci Res. 2008; 4(12):1708–1721.

[34] Embaby AM, Heshmat Y, Hussein A, Marey HS.A Sequential Statistical Approach towards an Optimized Production of a Broad Spectrum Bacteriocin Substance froma Soil Bacterium *Bacillus* sp. YAS 1 Strain. The World Sci J. 2014; 2014: 1–16. 10.1155/2014/396304 PMC4295142 25614886

[35] Embaby AM, Marey HS, Hussein A. Statistical-Mathematical Model to Optimize Chicken Feather Waste Bioconversion via *Bacillus licheniformis* SHG10: A Low Cost Effective and Ecologically Safe Approach. J Bioprocess Biotech. 2015; 5:231.

[36] Manderson K, Pinart M, Tuohy KN, Grace WE, Hotchkiss AT, Widmer W, Yadhav MP, Gibson GR, Rastall RA. *In Vitro* Determination of Prebiotic Properties of Oligosaccharides Derived from an Orange Juice Manufacturing By-Product Stream. Appl Env Microbiol. 2005; 71(12): 8383–8389. 16332825 10.1128/AEM.71.12.8383-8389.2005 PMC1317361

[37] Martínez M, Gullón HA, Schols HA, Alonso JL, Parajó JC. Assessment of the production of oligomeric compounds from sugarbeet pulp. Ind Eng Chem Res. 2009; 48: 4681–4687.

[38] Dung VK, Sam NTX, Huong NTT, Thu DT. Conditions of limited hydrolysis producing pectic oligosaccharides (POS) of high concentration passion fruit pectin. Tap Chi Sinh Hoc. 2015; 37(1),

[39] Olano-Martin E, Mountzouris KC, Gibson RG, Rastall RA. Continuous Production of Pectic Oligosaccharides in an Enzyme Membrane Reactor. J Food Sci. 2001; 66(7);966–971.

[40] Kühnel S, Hinz SWA, Pouvreau L, Wery J, Schols HA, Gruppen H. *Chrysosporium lucknowense* arabinohydrolasees effectively degrade sugarbeet arabinan. Bioresour Technol. 2010; 101: 8300–8307. 10.1016/j.biortech.2010.05.070 20566287

[41] Lama-Munoz A, Rodriguez-Gutiérrez G, Rubio-Senent F, Fernández-Bolanos J. Production, characterization and isolation of neutral and pectic oligosaccharides with low molecular weights from olive by-products thermally treated. Food Hydrocolloids.2012; 28: 92–104.

[42] Leijdekkers AGM, Bink JPM, Geuthes S, Schols HA, Gruppen H. Enzymatic saccharification of sugarbeet pulp for the production of galacturonic acid and arabinose; a study on the impact of the formation of recalcitrant oligosaccharides. Bioresour Technol. 2013; 128: 18–25. 10.1016/j.biortech.2012.10.126 23202377

